# Re-evaluation of dietary interventions in rheumatoid arthritis: can we improve patient conversations around food choices?

**DOI:** 10.1007/s00296-024-05541-4

**Published:** 2024-02-20

**Authors:** Prakriti Sharma, Shannon Brown, Elke M. Sokoya

**Affiliations:** 1 College of Medicine and Public Health, Flinders University, Flinders Health and Medical Research Institute, Adelaide, SA Australia; 2 Flinders University Library, Adelaide, SA Australia

**Keywords:** Autoimmune, Diet, Fasting, Food sensitivity, Rheumatoid arthritis

## Abstract

Rheumatoid arthritis (RA) is one of over 100 different types of autoimmune conditions. In RA, the cells of the immune system attack the tissue lining the joints, triggering inflammation. A large body of research suggests that the underlying trigger(s) of RA are unique to an individual. For example, increased risk of RA can be driven by smoking tobacco in one individual and mercury exposure in another. Due to the development of next-generation sequencing technology, the critical role of the microbiota in shaping RA risk has been elucidated. Therefore, it is surprising that diet, arguably the most important lever in shaping the gut microbiota, is ineffective in the treatment of RA, even in a sub-set of patients. To attempt to rationalise this apparent paradox, we conducted an umbrella review to address the question as to whether diet can affect outcomes in RA.

**Supplementary Information:**

The online version contains supplementary material available at 10.1007/s00296-024-05541-4.

## Introduction

Rheumatoid arthritis (RA) is an autoimmune disease characterised by systemic inflammation that primarily involves bilateral symmetric joint inflammation, resulting in joint pain. Left untreated, it progresses to cartilage and bone destruction. Symptoms, however, are not confined to the joints, and may include the heart, lungs, kidneys, skin, and eyes [1]. Therefore, RA is a systemic disease and any organ may be susceptible to tissue damage.

The manifestation of chronic systemic inflammation can arise from disruption of the microbiota [2]. This community of microbes includes trillions of bacteria, viruses, fungi, and archaea. In health, these microbes work together to assist in nutrient absorption and maintenance of oral tolerance. However, dysbiosis can lead to the release of harmful bacterial toxins such as lipopolysaccharide (LPS) that fuel intestinal inflammation and disrupt the intestinal epithelial barrier, thereby providing a gateway for the LPS to move into the systemic circulation. Gastrointestinal symptoms, including nausea, acid reflux, and abdominal pain, are common in RA [3] and RA patients often exhibit disruption to the normal well-balanced microbial ecosystem [4] along with increased immune cell infiltration within the lamina propria [5] that indicates disruption of the intestinal epithelial barrier.

Given the body of work demonstrating the impact of nutrition on the gut microbiota [6], the role of food choices on chronic disease risk [7], and the prevalence of dysbiosis in RA [4], one might speculate that dietary interventions should be effective in the treatment of RA. A preliminary PubMed search for the existing systematic reviews on the topic, however, revealed that there is only moderate evidence for a small benefit of certain dietary components in improving outcomes [8]. Therefore, the current umbrella review was performed, aiming to rationalise this apparent paradox and to identify strategies of interest for future research.

## Methods

### Search strategy

An initial limited search of MEDLINE (via Ovid) and CINAHL (via EBSCO) was performed to identify articles on the topic. The identified keywords and index terms were used to develop a complete search strategy in MEDLINE using all found appropriate search terms and Medical Subject Headings (MeSH). The search results were inspected to ensure the identification of relevant articles. The search strategy, including all relevant keywords and index terms, was adapted for the bibliographic databases MEDLINE (Ovid), CINAHL (EBSCOhost), Cochrane CENTRAL, and Scopus (Elsevier). The searches were run on 3 March, 2023 (Online Resource Table 1–4).

### Inclusion and exclusion criteria

The following inclusion criteria were used:

- systematic review with or without meta-analysis
- adults with RA
- assessing the effectiveness of dietary interventions (e.g., fasting, Mediterranean, vegetarian, and ketogenic) in RA
- published from 1 January, 2019 to present
- English language.

The following exclusion criteria were used:

- assessing the effectiveness of specific foods (e.g., almonds and blueberries) or specific nutrients (e.g., probiotics, omega-3 fatty acids, curcumin, and vitamin D)
- assessing whether diet increases risk of developing RA
- animal studies.

### Search terms

The search terms for each database are provided in the Online Resource (Table 1–4).

### Study selection

The search results were imported into Covidence [9] for duplicate removal and screening. The title and abstracts of the systematic reviews identified by the search were reviewed independently by two reviewers (EMS and PS) using the inclusion and exclusion criteria. Full-text articles were located and imported into Covidence and two reviewers (EMS and PS) independently assessed their final inclusion eligibility, based on the inclusion and exclusion criteria. Conflicts between the researchers in title and abstract and full-text screening for final inclusion were resolved by a discussion and the agreed decision was then cast by EMS.

### Quality assessment

The CASP Systematic Review checklist [10] was used to assess the quality of the extracted systematic reviews.

## Results

**Fig. 1:**
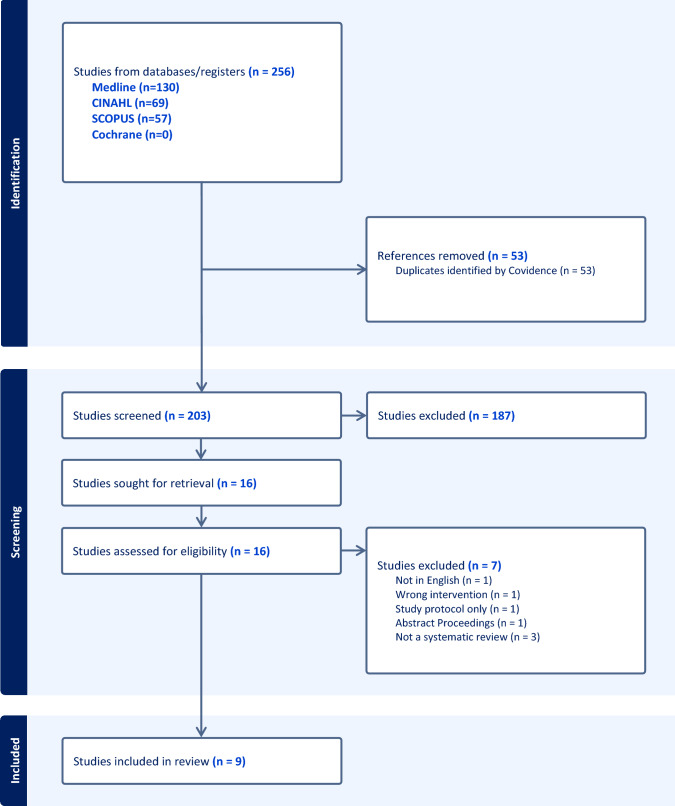
PRISMA flowchart providing a detailed overview of the study selection process

The Preferred Reporting Items for Systematic Reviews and Meta-analyses (PRISMA) flowchart providing a detailed overview of the study selection process can be found in Fig. 1. The search identified 256 articles in total (130 articles from Medline, 69 articles from CINAHL, 57 articles from SCOPUS, and 0 articles from Cochrane). Following upload into Covidence, 53 duplicate records were removed. Following title and abstract screening, 187 articles were deemed irrelevant, based on the inclusion and exclusion criteria, therefore leaving 16 articles. Following full-text review, seven articles were excluded, resulting in nine systematic reviews to be included in the present umbrella review.

### Synthesis of data

A summary of the systematic reviews assessing the effectiveness of dietary interventions in RA published from January 2019 is provided in the Online Resource (Table 5).

## Discussion

We have performed an umbrella review to understand the impact of food on the development of RA. As described in current clinical guidelines [8], it is true that not all RA patients benefit from dietary intervention; however, following consideration of the research data, a sub-set of RA patients appear to respond favourably to dietary changes. The dietary interventions providing the most significant effect are fasting, gluten-free vegan diets, and/or a customised dietary reintroduction protocol. Although the studies are heterogeneous with regards to the fasting protocol, the time of fast and inclusion criteria for medication use, fasting appears to show the most consistent improvements in both subjective [11–13] and objective [13–19] outcomes measures. At the end of a 1 week fast, significant improvements in objective measures, such as ESR, CRP, and IL-6, have been documented [13, 18–20]. This aligns with significant improvements in disease activity scores [13, 18–22], suggesting that dietary factors may be a source of inflammation in RA. In support of this body of research, a recent study has shown that removal of the gut microbiota with bowel cleansing followed by a 7-day fasting protocol in RA patients, led to a significant decline in DAS-28 scores, and markers of inflammation and mucosal barrier disruption [23].

While short-term fasting appears to have positive effects on certain outcome measures in a cohort of RA patients, these biomarkers return to baseline when regular diet is restored. Based on the existing studies, the most favourable results appear when the fasting period is followed by a gluten-free lacto-vegetarian diet and an individually adjusted reintroduction of food protocol. In a series of articles published by Kjeldsen-Kragh’s research group, the protocol comprised of introducing patients to a new food item every other day after the fasting period [14–16]. If RA symptoms were exacerbated within 2–48 h following food consumption, the food was omitted from the diet for at least 7 days. If RA symptoms were exacerbated upon the second re-entry, the food item was excluded for the remainder of the study period. Additionally, salt, strong spices, preservatives, tea, and coffee were avoided and patients were supplemented with either cod liver oil or vitamin D for the first four months. This personalised approach has been shown to improve short-term objective and subjective outcome measures [14] and were even sustained in some patients at 1 [16] and 2 years later [15]. Moreover, this protocol has reported significant changes in leucocyte count, IgM RF and complement C3 and C4 [16], suggesting that improvements in patient-reported outcomes were unlikely due to a placebo effect.

The benefits of this personalised food protocol were also documented by two other research groups [17, 24]. Beri et al*.* [24] omitted a fasting phase but placed RA patients on a stepwise dietary protocol. If RA symptoms were exacerbated, then the food item was eliminated from the diet. In this study, 10/14 participants demonstrated significant clinical improvement [24]. Darlington’s study also applied a 6-week dietary reintroduction protocol and significant improvements in a range of objective and subjective measures were observed [17]. The positive effect of eliminating particular food items has also been documented in case studies—in one case, the food item was dairy [25], and in the other, it was corn [26].

In a number of other studies, a “lacto-vegetarian diet” lacking meat and eggs following the fasting period has been trialled. Skoldstam and colleagues introduced this diet for 9 weeks following a 7–10 day fasting period and compared it to a normal omnivorous diet. Although subjective clinical measures were improved in a sub-group at the end of the fasting period, this was not sustained at the end of the lacto-vegetarian diet [27]. Fraser et al*.* showed that objective clinical outcomes (CRP, ESR, and IL-6) were improved during fasting but returned to baseline after 2 weeks on a lacto-vegetarian diet [18, 19]. Peltonen et al*.* followed a 7–10 day fast with a 3.5 month gluten-free vegan diet and a 9-month lacto-vegetarian diet. In the lacto-vegetarian diet, patients were allowed to reintroduce milk products; however, this was a personalised protocol where the food was removed if RA symptoms were exacerbated [22]. In this study, the researchers demonstrated that there was a sub-group of RA patients who showed significant clinical improvement and this aligned with changes within intestinal flora. These data suggest that it is not the lacto-vegetarian diet per se that elicits a beneficial effect, but perhaps the removal of gluten that reduces RA symptoms. Indeed, Hafström et al*.* trialled a gluten-free vegan diet for 1 year without fasting [28]. 41% of patients (9 out of 22) fulfilled the ACR20 improvement criteria, and in these responders, IgG antibody levels to gliadin and beta-lactoglobulin were significantly reduced. This data provides evidence that first fasting per se is not required to see a beneficial effect of dietary interventions, and second, some RA patients may be adversely affected by gluten-containing foods. Interestingly, a recent study in well-established RA patients reported significantly lower levels of inflammatory markers and pain following a three-month diet free of meat, lactose, and gluten [29]. However, given the emerging body of research around the importance of protein in supporting optimal metabolic health [30], it would be valuable to assess a gluten-free, dairy-free whole foods diet versus a regular whole foods diet. A caveat would be to ensure that the source of animal-based protein is not fed gluten-containing grains in the gluten-free cohort.

The clinical results described above suggest that food hypersensitivity may be involved in the pathogenesis of RA in certain patients. Indeed, a proportion of RA patients have significantly elevated serum IgA, IgG, and/or IgM antibodies directed against food proteins [28, 31–35]. O’Farrelly et al*.* demonstrated that 57% of RA patients had significantly raised levels of IgG antibodies to wheat and/or milk proteins [32]. Hvatum et al*.* [35] also reported significant elevations of IgG and IgM gliadin and casein antibodies within jejunal perfusion fluid from RA patients compared to controls. Kjeldsen-Kragh et al*.* [33] found that all patients had elevated (above the 90th percentile of healthy control measurements) IgA, IgG, or IgM activity against at least one dietary antigen. Using newer technology, Yang and colleagues [36] reported that 83% of RA patients were positive for at least one wheat protein antibody. Importantly, a decrease in gliadin and β-lactoglobulin was observed in the sub-group of RA patients who responded to a GF-vegan diet [28]. These data suggest that the translocation of food-derived peptides may occur in a proportion of RA patients, leading to activation of the immune system and antibody production. The positive effect of a GF-vegan diet in a sub-group of patients may be due to a diminished immune response to exogenous food antigens.

Taken together, the body of research suggests that antigenic load is higher in RA patients compared to the control population. A sub-group of RA patients respond favourably to fasting and these positive effects are maintained when an individual is placed on a gluten-free and/or a personalised food reintroduction protocol. These findings suggest that fasting, by removing the offending foods, can improve RA symptoms in certain individuals. While there appears to be individual variation in response to dietary items, as reflected by the personalised food reintroduction protocol, gluten does appear to be a major trigger. Rather than using a trial-and-error approach, measuring circulating levels of antibodies towards dietary-derived peptides may offer a strategy for identifying offending food products. In this way, the source of inflammation could be removed for a period of time, providing an opportunity for restoration of eubiosis, and fortitude of the intestinal barrier.

A recent systematic review and meta-analysis concluded that diets enriched with plant foods, such as the Mediterranean, vegetarian, and vegan diets, improve pain-reported outcomes [37]. Sköldstam et al*.* performed a randomised-controlled study comparing the MD diet with a control diet and reported significant reductions in DAS28, pain VAS, CRP, and platelet count [38]. Taken together, these studies suggest that plant-rich diets appear to confer some benefit likely mediated through positive impacts on the microbiota.

### The microbiota in health and RA

In health, beneficial bacteria outnumber pathogenic bacteria and, through their fermentation of complex resistant carbohydrates, they produce short-chain fatty acids (SCFAs), such as butyrate, acetate, and propionate [39, 40]. Indeed, consumption of a plant-rich diet or omega-3 rich diet has been correlated with higher levels of SCFA-producing bacteria and SCFAs [41–43]. SCFAs contribute to health by way of their anti-inflammatory properties, directly impacting the immune system by increasing colonic Treg production and suppressing Th17 cells, strengthening intestinal barrier function and decreasing bacterial translocation, facilitating mineral absorption, participating in glycemic control and stimulating mitochondrial biogenesis [44]. In addition to SCFAs, bacteria produce other metabolites including p-cresol, p-cresyl-glucuronide (pCG), indoxyl sulphate (IS), indole-3 acetic acid (IAA), and hydrogen sulphide, and trimethylamine N-oxide (TMAO) that may result in either protective or harmful effects on the intestinal barrier [45]. Beneficial bacteria also instruct the dendritic cells to produce immunoglobulin A (IgA)-secreting plasma cells and IgA, in turn, regulates the composition and function of the microbiota [46].

A growing body of research has reported differences in the gut microbiota of preclinical [47] and established [48–50] RA compared to healthy controls. Specifically, stool samples have revealed decreased gut microbial diversity in RA patients compared to healthy controls [49, 50]. In some patients, an expansion of certain pro-inflammatory bacterial species is associated with the development of RA. These bacteria include *Proteus mirabillis* [51], *Prevotella copri* [48], *Escherichia coli* [52], and *Klebsiella pneumoniae* [53]. In some individuals, the overgrowth may be within the small intestine, causing small intestinal bacterial overgrowth (SIBO). Individuals with SIBO often harbour bacteria that are commonly found in the large intestine, including the Gram-negative, carbohydrate fermenting microbes such as *E. coli*, *K. pneumonia,* and *Proteus mirabilis* [54–56], and this will likely impact the delicate intestinal epithelial barrier, as described below. In other RA patients, a depletion in beneficial commensal bacteria has been reported. For example, fewer bacteria belonging to the *Faecalibacterium* genus [50, 57] and the *Bacteroidetes* phyla [58] have been reported in RA patients. These bacteria produce the SCFA butyrate that exhibits anti-inflammatory properties and helps in maintaining the integrity of the gut epithelial barrier, as described below. In animal studies, SCFAs have been shown to inhibit the onset of arthritis by modulating IL-10 [5] and recent clinical work has shown that progression to the clinical phase of RA is correlated with lower levels of SCFAs [59]. Remarkably, a recent case report has described successful treatment of an RA patient with fecal microbiota transplantation [60].

### The mucosal barrier in health and RA

The gut microbiota helps maintain the integrity of the mucosal interface, comprising a mucous layer, a monolayer of columnar epithelial cells, and the gut-associated lymphoid tissue (GALT). The mucous layer consists mainly of large glycoproteins called mucins that shield the lamina propria from the intestinal contents. Mucin production is stimulated by Goblet cells (specialised secretory cells of the epithelial layer), cytokines, and SCFAs that are produced by the commensal bacteria [61]. The critical role of the microbiota in supporting the mucosal layer has been demonstrated in germ-free mice that display a significantly reduced mucosal layer [62]. A close association between the mucin layer and the immune response has also been demonstrated. In knock-out mice where the production of key proteins responsible for mucous secretion was inhibited, a significant increase in lymphocytes and inflammatory cytokines was observed, thereby highlighting the influence of mucosal layer integrity on inflammation [63].

The monolayer of epithelial cells is physically connected by tight junctions, adherens junctions, and desmosomes. The tight junction and adherens junction are comprised of cytoplasmic proteins [zonula occludens (ZO) family proteins and p120 catenin proteins], that are anchored to the actin cytoskeleton, and transmembrane proteins (occludin, claudin family proteins, and E-cadherin) [64]. Acting as a dynamic gate, the tight and adherens junctions maintain the integrity of the tight junctions and regulate the passive diffusion of ions and small water soluble solutes through the paracellular space.

The GALT, including the Peyer’s patches of the small intestine and thousands of isolated lymphoid follicles, is comprised of a variety of immune cells, including B cells, T cells, dendritic cells, and neutrophils. The GALT plays a vital role in the regulation of the host’s immune system through its interactions with the gut microbiota. It is responsible for both the recognition and initiation of the immune response through antigen sampling of naive B and T cells and the destruction of pathogenic components of the microbiota through the secretion of immunoglobulins into the mucosa [65]. Dendritic cells are involved in oral tolerance, either by penetrating the basal lamina and epithelial tight junctions to sample antigens from the mucosal surface or by interacting with antigens after they have crossed the epithelial layer or indirectly after their transcytosis across epithelial cells [66].

In health, most dietary proteins cross the intestinal epithelial barrier via the transcellular route, undergoing endocytosis and passage through the intestinal epithelial cell. This allows dietary proteins to be converted into smaller peptides by lysosomal degradation, preventing immune system activation. Some protein and carbohydrates, however, can resist digestion [67], thereby eliciting a non-IgE mediated immune response that causes intestinal inflammation and opening of the mucosal barrier, allowing pathologic levels of intact proteins to reach the small intestine [68]. A variety of factors can increase intestinal permeability including dysbiosis [69], non-steroidal anti-inflammatory drugs [70], alcohol [71], food additives [72], undigested gluten (gliadin protein) [73], and dietary lectins derived from legumes and cereal grains [74]. Digestive enzymes are fundamental to breaking down macronutrients such as protein and carbohydrates into their constituent amino acids and glucose, respectively. Interestingly, reduced gastric acid output has been shown in 80% of untreated RA patients that may incite reduced protease capacity [75] and enzyme therapy has been a considered approach in some patients with rheumatic disorders [76].

Abnormalities of the intestinal mucosa have been reported in a proportion of RA patients as evidenced by significantly elevated gastrointestinal leucocyte staining [77, 78] and increased intestinal permeability using labelled chromium [79]. Porzio et al*.* [80] documented ultrastructural changes within the intestinal mucosa of around 45% of their RA sample population. These changes included destruction of the brush border with loss of microvilli density, an increase in lysosome number, mitochondrial damage, and cell apoptosis. More recently, a body of research lends further support to the idea that mucosal barrier permeability is disturbed in RA [5, 81, 82].

### The gut–joint axis

The opening of the mucosal barrier provides an entry point where substances from the gut lumen can infiltrate the lamina propria. These substances include dietary peptides, toxins released from endogenous flora, such as lipopolysaccharides and peptidoglycans, and tight junction proteins (zonulin and actomyosin). Once in the lamina propria, they can trigger the immune system, leading to release of pro-inflammatory cytokines. Following a breakdown in immune tolerance, IgA and then IgG antibodies against food antigens can be produced in some individuals [83], resulting in persistent immunological stimulation [84]. Dietary peptide fragments, including those from milk, wheat, and legume proteins, have significant amino acid homologies with collagenous tissues found within the synovium [85, 86]. Due to this “molecular mimicry”, dietary peptide antibodies can target extra-intestinal sites such as the synovial joints.

Significant elevations of circulating LPS [82] and LPS activity [87] have been reported in RA patients. Animal studies have shown that LPS exacerbates the production of inflammatory cytokines and autoantibodies [88]. LPS can also activate the complement system [89] and bind to plasma proteins such as fibrinogen [90] that together could promote hypercoagulation [91]. A case for LPS driving RA etiology has been reviewed in more detail by Pretorius and colleagues [92].

Microbial products originating in the gut can move through the bloodstream and target the joints. Indeed, staining for peptidoglycan–polysaccharide complexes within synovial tissue has been reported in 16 out of 26 RA patients [93]. Moreover, in animal studies, neutralisation of circulating peptidoglycans has been shown to halt the development of arthritis [94].

### Future studies

In health, there are checkpoints in which self-antigen recognizing cells are deleted or inactivated, a process called tolerance. Chronic inflammation, however, can cause a breakdown of immune tolerance whereby immune cells become responsive to self-antigens [95], leading to an autoimmune disease such as RA. To treat and potentially reverse RA, identifying the source of chronic inflammation will be critical. Future studies may consider an approach by which immune reactivity towards dietary peptides is assessed whereby positive results likely suggest that a temporary exclusion diet may be an effective strategy of restoring health. Assessment of LPS antibodies would also be beneficial as elevation of this biomarker suggests dysbiosis and disruption of the mucosal barrier. A positive diagnosis could incorporate the introduction of a medically tailored diet and/or probiotics that serve to restore eubiosis and repair the mucosal barrier.

In individuals who do not show elevations in antibodies towards food-derived peptides, one could look towards other potential drivers of immune system activation such as synthetic toxins or pathogens. Cigarette smoke, a common synthetic toxin, is known to create dysbiosis [96] and increase risk of RA [97]. In other individuals, *Helicobacter pylori* (*H. pylori*) infection can drive dysbiosis [98] and increase risk of developing RA, particularly in women under the age of 30 [99]. Importantly, *H. pylori* treatment has been shown to significantly reduce inflammatory markers and improve clinical outcome measures [100].

## Conclusion

It is particularly important for rheumatologists to be aware of the specific research studies regarding dietary interventions in RA. To this end, the current review has highlighted the profound interaction between food, the microbiota, and the immune system as the source of inflammation in some RA patients. While eating to optimise the health of the microbiota appears to be helpful, the source of inflammation should also be identified. This requires a personalised approach that unfortunately cannot be described within the current framework of clinical practice guidelines that does not account for precision medicine. Ultimately, raised levels of antibodies to dietary proteins may select RA patients who would benefit from dietary intervention and a gut repair protocol. While we wait for diagnostic tests to be embedded into clinical practice to help guide dietary advice, RA patients can trial the effectiveness of a food elimination diet. In individuals where elevations towards dietary proteins are absent, one could look towards other potential drivers of immune system activation, such as toxins or pathogens.

### Supplementary Information

Below is the link to the electronic supplementary material.

Supplementary file1 (DOCX 103 KB)

## Data Availability

The supply of raw data is available upon request.
