# Supplementary material for: Re-evaluation of dietary interventions in rheumatoid arthritis: can we improve patient conversations around food choices?
Source: Rheumatol Int. 2024 Feb 20;44(8):1409–19. doi: 10.1007/s00296-024-05541-4 (PMC11222287; doi:10.1007/s00296-024-05541-4)
Supplement: Supplementary file 1 — Supplementary file1 (DOCX 103 KB) [file 296_2024_5541_MOESM1_ESM.docx]

**ONLINE RESOURCES**

**Table 1** Search syntax for Medline. Date search conducted 3 March, 2023.

| 1. # | 1. Query | 1. Results |
| --- | --- | --- |
| 1. 1 | 1. arthritis, rheumatoid/ or caplan syndrome/ or felty syndrome/ or rheumatoid nodule/ or rheumatoid vasculitis/ or sjogren's syndrome/ or still's disease, adult-onset/ | 1. 124422 |
| 1. 2 | 1. (arthritis adj2 rheumat*).tw,kf. | 1. 125925 |
| 1. 3 | 1. (felty* adj2 syndrome).tw,kf. | 1. 769 |
| 1. 4 | 1. (caplan* adj2 syndrome).tw,kf. | 1. 142 |
| 1. 5 | 1. rheumatoid nodule*.tw,kf. | 1. 926 |
| 1. 6 | 1. rheumatoid vasculitis.tw,kf. | 1. 402 |
| 1. 7 | 1. (sjogren* adj2 syndrome).tw,kf. | 1. 18058 |
| 1. 8 | 1. still* disease.tw,kf. | 1. 2860 |
| 1. 9 | 1. or/1-8 | 1. 170967 |
| 1. 10 | 1. Diet/ or exp diet therapy/ or exp Fasting/ | 1. 274197 |
| 1. 11 | 1. diet*.tw,kf. | 1. 675273 |
| 1. 12 | 1. ((energy or calor* or "kilo-joule" or kilojoule or "kilo joule") adj2 (intake or restrict*)).tw,kf. | 1. 48567 |
| 1. 13 | 1. fast*.tw,kf. | 1. 638253 |
| 1. 14 | 1. ((food or nutrit*) adj3 (habit* or intake or restrict*)).tw,kf. | 1. 75681 |
| 1. 15 | 1. or/10-14 | 1. 1402758 |
| 1. 16 | 1. (systematic review or meta-analysis).pt. | 1. 302969 |
| 1. 17 | 1. meta-analysis/ or systematic review/ or systematic reviews as topic/ or meta-analysis as topic/ or "meta analysis (topic)"/ or "systematic review (topic)"/ or exp technology assessment, biomedical/ or network meta-analysis/ | 1. 340610 |
| 1. 18 | 1. ((systematic* adj3 (review* or overview*)) or (methodologic* adj3 (review* or overview*))).tw,kf. | 1. 305509 |
| 1. 19 | 1. ((quantitative adj3 (review* or overview* or synthes*)) or (research adj3 (integrati* or overview*))).tw,kf. | 1. 15068 |
| 1. 20 | 1. ((integrative adj3 (review* or overview*)) or (collaborative adj3 (review* or overview*)) or (pool* adj3 analy*)).tw,kf. | 1. 37594 |
| 1. 21 | 1. (data synthes* or data extraction* or data abstraction*).tw,kf. | 1. 38832 |
| 1. 22 | 1. (handsearch* or hand search*).tw,kf. | 1. 10941 |
| 1. 23 | 1. (mantel haenszel or peto or der simonian or dersimonian or fixed effect* or latin square*).tw,kf. | 1. 34615 |
| 1. 24 | 1. (met analy* or metanaly* or technology assessment* or HTA or HTAs or technology overview* or technology appraisal*).tw,kf. | 1. 11862 |
| 1. 25 | 1. (meta regression* or metaregression*).tw,kf. | 1. 13957 |
| 1. 26 | 1. (meta-analy* or metaanaly* or systematic review* or biomedical technology assessment* or bio-medical technology assessment*).mp,hw. | 1. 449628 |
| 1. 27 | 1. (medline or cochrane or pubmed or medlars or embase or cinahl).ti,ab,hw. | 1. 327969 |
| 1. 28 | 1. (cochrane or (health adj2 technology assessment) or evidence report).jw. | 1. 21262 |
| 1. 29 | 1. (comparative adj3 (efficacy or effectiveness)).tw,kf. | 1. 17118 |
| 1. 30 | 1. (outcomes research or relative effectiveness).tw,kf. | 1. 11043 |
| 1. 31 | 1. ((indirect or indirect treatment or mixed-treatment or bayesian) adj3 comparison*).tw,kf. | 1. 4238 |
| 1. 32 | 1. (multi* adj3 treatment adj3 comparison*).tw,kf. | 1. 288 |
| 1. 33 | 1. (mixed adj3 treatment adj3 (meta-analy* or metaanaly*)).tw,kf. | 1. 178 |
| 1. 34 | 1. umbrella review*.tw,kf. | 1. 1328 |
| 1. 35 | 1. (multi* adj2 paramet* adj2 evidence adj2 synthesis).tw,kf. | 1. 13 |
| 1. 36 | 1. (multiparamet* adj2 evidence adj2 synthesis).tw,kf. | 1. 18 |
| 1. 37 | 1. (multi-paramet* adj2 evidence adj2 synthesis).tw,kf. | 1. 11 |
| 1. 38 | 1. or/16-37 | 1. 659425 |
| 1. 39 | 1. 9 and 15 and 38 | 1. 130 |

1. **Table 2** Search syntax for CINAHL. Date search conducted 3 March, 2023.

| # | Query | Results |
| --- | --- | --- |
| S17 | S8 AND S15 AND S16 | 69 |
| S16 | (MH "meta analysis" OR MH "systematic review" OR MH "Technology, Medical/EV" OR PT "systematic review" OR PT "meta analysis" OR (((TI systematic* OR AB systematic*) N3 ((TI review* OR AB review*) OR (TI overview* OR AB overview*))) OR ((TI methodologic* OR AB methodologic*) N3 ((TI review* OR AB review*) OR (TI overview* OR AB overview*)))) OR (((TI quantitative OR AB quantitative) N3 ((TI review* OR AB review*) OR (TI overview* OR AB overview*) OR (TI synthes* OR AB synthes*))) OR ((TI research OR AB research) N3 ((TI integrati* OR AB integrati*) OR (TI overview* OR AB overview*)))) OR (((TI integrative OR AB integrative) N3 ((TI review* OR AB review*) OR (TI overview* OR AB overview*))) OR ((TI collaborative OR AB collaborative) N3 ((TI review* OR AB review*) OR (TI overview* OR AB overview*))) OR ((TI pool* OR AB pool*) N3 (TI analy* OR AB analy*))) OR ((TI "data synthes*" OR AB "data synthes*") OR (TI "data extraction*" OR AB "data extraction*") OR (TI "data abstraction*" OR AB "data abstraction*")) OR ((TI handsearch* OR AB handsearch*) OR (TI "hand search*" OR AB "hand search*")) OR ((TI "mantel haenszel" OR AB "mantel haenszel") OR (TI peto OR AB peto) OR (TI "der simonian" OR AB "der simonian") OR (TI dersimonian OR AB dersimonian) OR (TI "fixed effect*" OR AB "fixed effect*") OR (TI "latin square*" OR AB "latin square*")) OR ((TI "met analy*" OR AB "met analy*") OR (TI metanaly* OR AB metanaly*) OR (TI "technology assessment*" OR AB "technology assessment*") OR (TI HTA OR AB HTA) OR (TI HTAs OR AB HTAs) OR (TI "technology overview*" OR AB "technology overview*") OR (TI "technology appraisal*" OR AB "technology appraisal*")) OR ((TI "meta regression*" OR AB "meta regression*") OR (TI metaregression* OR AB metaregression*)) OR (MW meta-analy* OR MW metaanaly* OR MW "systematic review*" OR MW "biomedical technology assessment*" OR MW "bio-medical technology assessment*") OR ((TI medline OR AB medline OR MW medline) OR (TI cochrane OR AB cochrane OR MW cochrane) OR (TI pubmed OR AB pubmed OR MW pubmed) OR (TI medlars OR AB medlars OR MW medlars) OR (TI embase OR AB embase OR MW embase) OR (TI cinahl OR AB cinahl OR MW cinahl)) OR (SO Cochrane OR SO health technology assessment OR SO evidence report) OR ((TI comparative OR AB comparative) N3 ((TI efficacy OR AB efficacy) OR (TI effectiveness OR AB effectiveness))) OR ((TI "outcomes research" OR AB "outcomes research") OR (TI "relative effectiveness" OR AB "relative effectiveness")) OR (((TI indirect OR AB indirect) OR (TI "indirect treatment" OR AB "indirect treatment") OR (TI mixed-treatment OR AB mixed-treatment) OR (TI bayesian OR AB bayesian)) N3 (TI comparison* OR AB comparison*)) OR ((TI multi* OR AB multi*) N3 (TI treatment OR AB treatment) N3 (TI comparison* OR AB comparison*)) OR ((TI mixed OR AB mixed) N3 (TI treatment OR AB treatment) N3 ((TI meta-analy* OR AB meta-analy*) OR (TI metaanaly* OR AB metaanaly*))) OR (TI "umbrella review*" OR AB "umbrella review*") OR ((TI multi* OR AB multi*) N2 (TI paramet* OR AB paramet*) N2 (TI evidence OR AB evidence) N2 (TI synthesis OR AB synthesis)) OR ((TI multiparamet* OR AB multiparamet*) N2 (TI evidence OR AB evidence) N2 (TI synthesis OR AB synthesis)) OR ((TI multi-paramet* OR AB multi-paramet*) N2 (TI evidence OR AB evidence) N2 (TI synthesis OR AB synthesis)) | 296,777 |
| S15 | S9 OR S10 OR S11 OR S12 OR S13 OR S14 | 312,997 |
| S14 | (MH "Fasting") | 7,892 |
| S13 | TI ( ((food OR nutrit*) N3 (habit* OR intake OR restrict*)) ) OR AB ( ((food OR nutrit*) N3 (habit* OR intake OR restrict*)) ) | 21,931 |
| S12 | TI fast* OR AB fast* | 87,003 |
| S11 | TI ( ((energy OR calor* OR "kilo-joule" OR kilojoule OR "kilo joule") N2 (intake OR restrict*)) ) OR AB ( ((energy OR calor* OR "kilo-joule" OR kilojoule OR "kilo joule") N2 (intake OR restrict*)) ) | 16,836 |
| S10 | TI diet* OR AB diet* | 156,256 |
| S9 | (MH "Diet+") | 140,977 |
| S8 | S1 OR S2 OR S3 OR S4 OR S5 OR S6 OR S7 | 39,461 |
| S7 | TI (sjogren* N2 syndrome) OR AB (sjogren* N2 syndrome) | 3,112 |
| S6 | TI rheumatoid vasculitis OR AB rheumatoid vasculitis | 130 |
| S5 | TI rheumatoid nodule* OR AB rheumatoid nodule* | 162 |
| S4 | TI (caplan* N2 syndrome) OR AB (caplan* N2 syndrome) | 4 |
| S3 | TI (felty* N2 syndrome) OR AB (felty* N2 syndrome) | 59 |
| S2 | TI (arthritis N2 rheumat*) OR AB (arthritis N2 rheumat*) | 26,510 |
| S1 | (MH "Arthritis, Rheumatoid+") | 31,407 |

1. **Table 3** Search syntax for Scopus.

| **Date** | **Search syntax** |
| --- | --- |
| 3 March, 2023 | 1. ( TITLE-ABS-KEY ( ( sjogren* W/2 syndrome ) OR "rheumatoid vasculitis" OR "rheumatoid nodule*" OR ( caplan* W/2 syndrome ) OR ( felty* W/2 syndrome ) OR ( arthritis W/2 rheumat* ) ) ) AND ( TITLE-ABS-KEY ( ( ( ( food OR nutrit* ) W/3 ( habit* OR intake OR restrict* ) ) OR fast* OR fast* OR ( ( energy OR calor* OR "kilo-joule" OR kilojoule OR "kilo joule" ) W/2 ( intake OR restrict* ) ) ) ) ) AND ( TITLE-ABS-KEY ( ( ( systematic* ) W/3 ( review* OR overview* ) ) OR ( methodologic* W/3 ( review* OR overview* ) ) OR ( ( quantitative ) W/3 ( review* OR overview* OR synthes* ) ) OR ( ( research ) W/3 ( integrati* OR overview* ) ) OR ( ( integrative ) W/3 ( review* OR overview* ) ) OR ( ( collaborative ) W/3 ( review* OR overview* ) ) OR ( ( pool* ) W/3 ( analy* ) ) OR "data synthes*" OR "data extraction*" OR "data abstraction*" OR handsearch* OR "hand search*" OR "mantel haenszel" OR peto OR "der simonian" OR dersimonian OR "fixed effect*" OR "latin square*" OR "met analy*" OR metanaly* OR "technology assessment*" OR hta OR htas OR "technology overview*" OR "technology appraisal*" OR "meta regression*" OR metaregression* OR meta-analy* OR metaanaly* OR "systematic review*" OR "biomedical technology assessment*" OR "bio-medical technology assessment*" OR medline OR cochrane OR pubmed OR medlars OR embase OR cinahl OR cochrane OR meta-analy* OR metaanaly* OR "outcomes research" OR "relative effectiveness" OR "evidence report" ( ( health ) W/2 "technology assessment" ) ) OR ( ( comparative W/3 ( efficacy OR effectiveness ) ) OR ( ( indirect OR "indirect treatment" OR mixed-treatment OR bayesian ) W/3 ( comparison* ) ) ) ) |

1. **Table 4** Search syntax for Cochrane CENTRAL

| **Date** | **Search syntax** |
| --- | --- |
| 3 March, 2023 | 1. (((sjogren* NEAR/2 syndrome) OR "rheumatoid vasculitis" OR "rheumatoid nodule*" OR (caplan* NEAR/2 syndrome) OR (felty* NEAR /2 syndrome) OR (arthritis NEAR/2 rheumat*)) AND (((food OR nutrit*) NEAR /3 (habit* OR intake OR restrict*)) OR fast* OR fast* OR ((energy OR calor* OR "kilo-joule" OR kilojoule OR "kilo joule") NEAR /2 (intake OR restrict*)))) |

**Table 5** Summary of systematic reviews (2019-2023) assessing the effectiveness of dietary interventions in RA.

| **Author, Year [Country]** | **Title** | **Author Findings** |
| --- | --- | --- |
| Gwinnutt *et al*, 2022 [1] [various – as members of the EULAR taskforce] | Effects of diet on the outcomes of rheumatic and musculoskeletal diseases (RMDs): systematic review and meta-analyses informing the 2021 EULAR recommendations for lifestyle improvements in people with RMDs | The authors considered the literature assessing the effect of diet regimens on RA. The search resulted in one meta-analysis [2]. Cramp *et al* included a subgroup analysis of one study [3] looking at the effect of a Mediterranean diet (MD) compared to control on fatigue in RA patients. Compared to baseline, the MD significantly improved vitality.  The search resulted in 12 RCTs [3, 4] [5-10] [11] [12, 13] [14].  Podas *et al* [4] enrolled 30 RA patients and randomly assigned them to either a 2-week elemental diet (n=21) or oral prednisolone (n=9). No control group was included. There was a significant improvement in subjective clinical parameters in both groups. ESR, CRP and hemoglobin only improved in the prenisolone group.  Skoldstam *et al* [3] randomly allocated RA patients (active for at least 2 years) to either the MD diet (n=26) or the control diet (n=25). Outcome measures were assessed at baseline, week 3, week 6 and week 12. At week 12, significant reductions in DAS28, pain VAS, CRP and platelet count were observed. Note that the difference between the two groups was notable only in the second half of the trial.  Hafström *et al* [5] randomly assigned RA patients to either a gluten-free vegan diet (n=38/22 completed) or a non-vegan diet (n=28/25 completed) for one year. 41% of patients (9 out of 22) fulfilled the ACR20 improvement criteria and IgG antibody levels to gliadin and beta-lactoglobulin were significantly reduced in the responders. The data provide evidence that **dietary modification maybe of clinical benefit to certain RA patients**.  Sarzi-Puttini *et al* [6] randomly assigned patients to either an experimental diet (n=25/22 completed) or to a control diet (n=25/ 21 completed) for 24 weeks. The experimental diet was high in mono and polyunsaturated fatty acids and low in saturated fatty acids with the elimination and/or restrictions of certain dietary items. The control diet was a typical well-balanced diet. **Significant improvement in Ritchie’s index, swollen joints and tender joints were observed with the experimental diet.**  Holst-Jensen *et al* [7] randomly assigned RA patients to either a liquid elemental peptide diet (n=12) or normal food (control; n=14) for 4 weeks. At 4-weeks, HAQ-score and average pain over last week were significantly reduced in the peptide diet group. Reduction in ESR and number of swollen joints did not reach statistical significance. These parameters returned to baseline at 3-months and 6-months when the normal diet had been restored. Only one patient was classified as a responder.  Nenonen *et al* [8] randomly assigned RA patients to either an uncooked vegan diet (n=19) or normal food (control; n=19). There was a **statistically significant improvement in rheumatic pain, joint swelling and morning stiffness in the intervention group** but no statistically significant effect on CRP, ESR, HAQ, and VAS.  Kavanagh *et al* [9] randomly assigned RA patients to either a partial elemental diet along with a small number of foods (n=24) or normal food (control; n=23) for 4-weeks. The additional foods that were added to the elemental diet were chicken, fish, rice, carrots, runner beans and bananas. The partial elemental diet was followed by re-introduction of foods, one at a time, at intervals no shorter than 2 days. **After 4-weeks, there was a significant increase in grip strength and weight loss and a significant decrease in CRP in the intervention group**.  Haugen *et al* [10] performed a double-blind controlled pilot study and randomly assigned RA patients to either an elemental diet (n=10) or control diet (n=7) for 3 weeks followed by a regular diet for 1 week. There was a **significant improvement in tender joint count for RA patients on the elemental diet**. 9 out of 10 RA patients on the elemental diet experienced diarrhea which indicates an altered bacterial flora. Unfortunately use of non-steroidal anti-inflammatory drugs (NSAIDs) is a confounder. Although the overall findings do not provide benefit of an elemental diet in RA patients, the study is likely underpowered, along with the use of NSAIDs that are known to disrupt the small intestinal epithelial barrier [15].  van de Laar [11] performed a double-blind randomised study where seropositive RA patients were assigned to either the allergen-free diet (n=45) or the hypoallergenic diet (n=49). In the first 4-weeks, patients consumed their regular diet. During the second 4-weeks, patients consumed their assigned diet. During the final 4-weeks, patients returned to their regular diet. Notably both arms of the study had reduced exposure to potential allergens. There were considerable differences in the response of individual patients to the dietary manipulation. Of the 94 participants, **nine patients demonstrated a 20% improvement during the 4-week diet challenge followed by a more than 20% deterioration when regular diet was resumed.**  Panush *et al* [12] performed a 10-week double blind placebo controlled randomised study. RA patients were randomly assigned to either an experimental diet (n=11) or placebo diet (n=15). Notably, the experimental diet was not an elemental diet. Rather, it consisted of a specific “popular” diet free of additives, preservatives, dairy, red meat, fruit and herbs and spices. The placebo diet consisted of meat (no turkey), eggs, fruit (except bananas and strawberries), white wine, additives and preservatives. There were no statistically significant differences between patients on the experimental and placebo diets. However 11 patients (5 on experimental diet and 6 on placebo diet) showed at least 16% average improvement in ESR, morning stiffness, walk time, grip strength, number of tender and number of swollen joints. These observations support the idea that **dietary manipulation may be beneficial for selected RA patients**.  Sundqvist *et al* [13] performed a randomised placebo-controlled trial. RA patients were randomly assigned to either the fasting group (n=5) or the placebo group (n=5) who continued with their regular diet. All patients were taking a daily NSAID. However during the 10-day fasting period, NSAIDs were stopped and only paracetamol was allowed. After the fasting period, patients were assigned to a lactovegetarian diet. Intestinal permeability was assessed with PEG-400. The permeability to PEG-400 was decreased after fasting, but then increased during the lactovegetarian diet. These data suggest that **dietary intake could be contributing to a leaky gut in RA patients**. In addition, after the fast, the clinical 6-joint score and the orosomucoid concentration were reduced.  Skoldstam *et al* [14] performed a 10-week randomised trial. RA patients were randomly assigned to either a fasting group for 7-10 days (n=15) or the control group (n=10). The fasting group were provided with fruit and vegetable juice only and twice daily water enemas. NSAIDs were not allowed during the fast. The fast was followed by a 9-week lactovegetarian diet. **At the end of the fast, there was a statistically significant reduction in pain, stiffness, Ritchie’s index, clinical 6-joint score and ring-size test.**  The search resulted in 5 non-randomised trials [16-20]. Abendroth *et al* [16] performed a non-randomised clinical trial where they enrolled 50 RA patients into either a 7-day fasting therapy (n=22) or a Mediterranean diet (n=28). This fasting period was followed by 2 days of a low calorie diet with stepwise reintroduction of foods. A normocaloric diet was reached again with the evening of study day 13. DAS-28 significantly decreased in both arms of the study. The VAS pain rating decreased in both groups which was significantly greater in the fasting arm. No negative control group (standard of care) was included.  McKellar *et al* [17] performed a clinical trial where they enrolled 130 female RA patients into either a Mediterranean diet (n=75) or the control (n=55). While randomisation was considered, purposive sampling was required due to the inherent nature of attending cooking classes within the Mediterranean diet group. **At six months, there was a significant difference in patient pain VAS score between groups**.  Adam *et al* [18] recruited 68 RA patients and assigned them to either an 8-month anti-inflammatory diet (AID; n=34) or normal Western diet (WD; n=34). Purposive sampling was used to ensure compliance with the AID diet. Patients in both arms of the study were randomised for allocation to placebo or fish oil for 3-months each in a double-blind cross-over study. Notably **after 1-month of the diet, CRP and immunoglobulin G were significant lower in the AID group compared to the WD group**.  Fraser *et al* [19] recruited 23 RA patients and assigned the first 10 patients to a 7-day supervised fasting protocol with a limited amount of vegetable juice (n=10) and the next 13 patients to the 7-day ketogenic protocol (n=13). This was followed by re-feeding (2-week lacto-vegetarian diet). **Fasting resulted in a significant decrease in CRP, ESR, IL-6 and tender joint score** which then returned to baseline after re-feeding.  Denissov *et al* [20] recruited 92 RA patients and assigned them to either an anti-inflammatory, hypoallergenic diet (n=68) or a control arm (n=24; standard-of-care; conventional anti-inflammatory therapy). In the **diet arm of the study, there were significant changes in arthralgia points, grip strength and pain. This was aligned with a significant reduction in CRP** (no change in ESR or fibrinogen).  Search resulted in 1 single arm study [21]. The authors recruited 24 RA patients and assigned them to a 4-week low-fat vegan diet. No control arm was included. Self-reported indices such as pain, ability to function and joint tenderness improved. At 4-weeks, CRP and ESR were unchanged.  Search resulted in 1 extension to an RCT comparing responders with non-responders [22]. The authors performed a two year follow-up from their original study where patients were randomised to either a 7-10 day fast followed by a gluten-free vegetarian diet for one year (n=27) or control diet (n=26) [23]. In this follow-up study, there were 22 participants (10 responders and 12 non-responders) in the GF vegetarian diet and 23 participants (2 responders and 21 non-responders) in the control arm. **The diet responders had a significant improvement in pain, tender joints and swollen joints**. The diet responders also showed a reduction in ESR but this did not reach statistical significance. There is a risk of a type II error due to the reduced sample size.  Gwinnutt *et al* conclude that “there is little evidence suggesting dietary factors can make large differences to the outcomes of people with RMDs [rheumatic and musculoskeletal diseases].” |
|  |  |  |
| Lanspa *et al*, 2022 [24] [USA] | A systematic review of nutritional interventions on key cytokine pathways in rheumatoid arthritis and its implications for comorbid depression: is a more comprehensive approach required? | The purpose of this systematic review was to identify nutritional patterns and dietary supplements that decrease markers of inflammation.  The main findings are:  1) poor diet quality and adherence to an anti-inflammatory diet in RA patients increase inflammatory markers and pain scores  2) dietary consumption of antioxidants, PUFAs and fiber have preferable disease outcomes in RA patients  3) flavonoid supplementation can reduce inflammatory cytokines in RA  4) probiotic supplementation may ameliorate the gut microbiota profile and reduce disease activity |
|  |  |  |
| Philippou *et al*, 2021 [25] [UK] | Rheumatoid arthritis and dietary interventions: systematic review of clinical trials | The purpose of this systematic review was to provide the current evidence regarding the effect of diet and supplements on RA outcomes. The authors conclude that fasting resulted in significant but transient improvements. Outcomes from vegetarian, elimination, peptide or elemental diets appear to be varied. The Mediterranean diet has shown improvements in some RA disease activity measures.  The authors conclude that “Some dietary approaches may improve RA symptoms and thus it is recommended that nutrition should be routinely addressed.” |
|  |  |  |
| Raad *et al*, 2021 [26] [Ireland] | Dietary interventions with and without omega-3 supplementation for the management of rheumatoid arthritis: a systematic review | The purpose of this systematic review was to assess the effect of dietary interventions, with and without omega-3, on RA outcomes. The authors conclude that “the evidence on diet and RA is insufficient and inconclusive” and “dietary interventions with an anti-inflammatory basis may be an effective way for adults with RA seeking complementary treatments, potentially leading to improvements in certain parameters.” |
|  |  |  |
| Schönenberger *et al*, 2021 [27] [Switzerland] | Effect of anti-inflammatory diets on pain in rheumatoid arthritis: a systematic review and meta-analysis | The authors performed a systematic review and meta-analysis to investigate the effect of potentially anti-inflammatory diets (Mediterranean, vegetarian, vegan, ketogenic) on pain in RA. The authors conclude that “all studies had a high risk of bias and the evidence was very low. The main conclusion is that anti-inflammatory diets resulted in significantly lower pain than ordinary diets. |
|  |  |  |
| Wagenaar *et al*, 2021 [28] [The Netherlands] | The effect of dietary interventions on chronic inflammatory diseases in relation to the microbiome: a systematic review | The aim of this systematic review was to determine the effect of dietary interventions, particularly fiber intake, on chronic inflammatory diseases (including RA) and the microbiome.  Four studies have assessed the effect of a high fiber diet on RA outcomes and intestinal microflora. Peltonen *et al* [29] recruited 53 RA patients into a 13-month study and randomly assigned them to either a diet group (n=27) or control group (n=26). The protocol for the diet group consisted of attendance to a health farm, a 7-10 day fast, followed by a 3.5 month vegan diet (gluten and citrus fruits were also excluded). After the fast, a new food item was added to the diet every second day. If an increase in RA symptoms were observed within 48 hours it was omitted for 7 days. If the reintroduction of the food item after 7 days re-exacerbated the symptoms, it was excluded from the diet for the remaining study period. This was followed by a lactovegetarian phase where patients were allowed to reintroduce milk, other dairy and gluten. However, if symptoms worsened, then a food item was removed as described previously. Notably, this approach of fasting followed by personalised reintroduction of food is similar to that of Kjeldsen-Kragh’s studies [22, 23, 30]. Significant differences in the fecal flora were found between the high improvement (blinded clinical evaluation) index group (H1) group and the low improvement (L1) group that suggests an association between intestinal bacteria and clinical improvement of RA.  Peltonen *et al* [31] then recruited 36 RA patients and randomly assigned them to either a one-month uncooked vegan diet group (n=18) or control group (n=18). 5/18 vegan diet patients demonstrated high improvement of clinical symptoms, versus 0/18 in the control group. The bacterial cellular fatty acid profile of stool samples was significantly different in the vegan diet group when comparing the pre-test with the test period samples and the post-test samples. No significant difference was seen in the control group. The findings suggest that the temporary clinical improvements imparted by the vegan diet maybe mediated through the gut microbiome changes.  Michalsen *et al* [32] enrolled patients with RA (n=16) or fibromyalgia (n=35) and participants were given a choice to start a mostly vegetarian diet (n=21) or an 8-day intermittent modified fasting therapy (n=30). The fast was followed by a two-day low calorie diet with stepwise reintroduction of food. Unfortunately, it is not clear as to whether food items were continued if RA symptoms were exacerbated. No significant changes were observed in the fecal counts, sIgA or stool pH in either of the treatment arms.  Abendroth *et al* [16] performed a non-randomised clinical trial where they enrolled 50 RA patients into either a 7-day fasting therapy (n=22) or a Mediterranean diet (n=28). The fasting period was followed by two days on a low calorie diet with stepwise reintroduction of foods. It is not stipulated whether patients could refrain from eating food if they noticed symptoms flare. DAS-28 significantly decreased in both arms of the study. The VAS pain rating decreased in both groups which was significantly greater in the fasting arm. There were no significant changes in laboratory variables. No negative control group (standard of care) was included.  The authors conclude that “Overall..high-fiber plant-based dietary interventions were consistently more effective at reducing disease-specific outcomes..” |
| Elma *et al*, 2020 [33] [Belgium] | Do nutritional factors interact with chronic musculoskeletal pain? A systematic review | The purpose of this systematic review was to assess the evidence on the potential link between diet and musculoskeletal pain (including RA, OA and fibromyalgia). It included nine experimental studies and three observational studies. The following three diets were considered in the analysis.  **(1) Vegetarian Diet**  An RCT including people with rheumatoid arthritis (n = 16) applied a lactovegetarian diet (no meat and eggs) for nine weeks following a 7-10 day fasting period compared to a normal omnivorous diet [14]. Participants were not allowed to consume animal or fish protein (including eggs), alcohol, tobacco, coffee, or tea. Intake of salt, sugar, white flour, fresh milk, and cream were also discouraged.  Although **5 people with RA (out of 16) showed a significant improvement in visual analogue scale (VAS) pain score compared to baseline measurements** after the fasting period, there was no statistically significant improvement in pain after the implementation of the lactovegetarian diet compared to the control group.  **(2) Vegan Diet**  An uncontrolled clinical trial including people with RA who changed their regular diet to a low-fat vegan diet for 4 weeks [21]. The low-fat vegan diet  contained no animal products or added fats and oils of any kind. **Compared to baseline measurements, the VAS pain score showed a significant improvement** in response to the low-fat vegan diet.  **(3) Elemental diet**  In an RCT, the effect of a (commercialised) liquid peptide diet on RA pain was investigated [7]. Daily caloric intake of the participants was adjusted to the 30 kcal/kg individually. Participants were not allowed to eat and drink anything else during the four week liquid peptide diet except for soda water. The **average VAS pain score improved significantly after 4 weeks of dietary intervention**, but this improvement disappeared at the three months follow-up from the end of the four weeks of intervention.  The authors concluded that “plant-based diets might have pain relieving effects on musculoskeletal pain.” |
|  |  |  |
| Genel *et al*, 2020 [34] [Australia] | Health effects of a low-inflammatory diet in adults with arthritis: a systematic review and meta-analysis | The authors considered five randomised trials and two prospective trials with either RA or osteoarthritis. Articles that were considered included two articles on OA [35] [36] and one article on RA [3].  The authors state that there was very low-quality evidence that a low-inflammatory diet results in a reduction in inflammatory biomarkers at 2-4 months. However, following a sub-group analysis based on diagnosis, the RA group had significant reductions in inflammatory biomarkers CRP, IL-6 and IL-16 at 2-4 months (P= 0.0004). No significant effects were found for physical function, general health, and joint pain.  The authors concluded that in inflammatory arthritis conditions, including RA, there is “very low level evidence suggest[ing] that low-inflammatory diets … compared to usual diets are associated with … improvement in inflammatory biomarkers”. |
|  |  |  |
| Nelson *et al*, 2020 [37] [Sweden] | Do interventions with diet or dietary supplements reduce the disease activity score in rheumatoid arthritis? A systematic review of randomized controlled trials | The aim of this systematic review was to assess whether diet or dietary supplements are able to reduce disease activity in RA. The authors reported a moderate strength of evidence for positive effects on disease activity in RA for Mediterranean diet, spices (ginger powder, cinnamon powder, saffron), antioxidants (quercetin and ubiquinone), and probiotics containing Lactobacillus Casei.  The authors concluded that there is “evidence that some dietary interventions may have positive effects on DAS-28”. |

**REFERENCE LIST**

1. Gwinnutt JM, Wieczorek M, Rodríguez-Carrio J, Balanescu A, Bischoff-Ferrari HA, Boonen A, Cavalli G, de Souza S, de Thurah A, Dorner TE, Moe RH, Putrik P, Silva-Fernández L, Stamm T, Walker-Bone K, Welling J, Zlatković-Švenda M, Guillemin F, Verstappen SMM (2022) Effects of diet on the outcomes of rheumatic and musculoskeletal diseases (RMDs): systematic review and meta-analyses informing the 2021 EULAR recommendations for lifestyle improvements in people with RMDs. RMD Open 8:e002167 <https://doi.org/10.1136/rmdopen-2021-002167>

2. Cramp F, Hewlett S, Almeida C, Kirwan JR, Choy EH, Chalder T, Pollock J, Christensen R (2013) Non-pharmacological interventions for fatigue in rheumatoid arthritis. Cochrane Database Syst Rev 8:CD008322 <https://doi.org/10.1002/14651858.CD008322.pub2>

3. Sköldstam L, Hagfors L, Johansson G (2003) An experimental study of a Mediterranean diet intervention for patients with rheumatoid arthritis. Ann Rheum Dis 62(3):208-14 <https://doi.org/10.1136/ard.62.3.208>

4. Podas T, Nightingale JM, Oldham R, Roy S, Sheehan NJ, Mayberry JF (2007) Is rheumatoid arthritis a disease that starts in the intestine? A pilot study comparing an elemental diet with oral prednisolone. Postgrad Med J 83(976):128-31 <https://doi.org/10.1136/pgmj.2006.050245>

5. Hafström I, Ringertz B, Spångberg A, von Zweigbergk L, Brannemark S, Nylander I, Rönnelid J, Laasonen L, Klareskog L (2001) A vegan diet free of gluten improves the signs and symptoms of rheumatoid arthritis: the effects on arthritis correlate with a reduction in antibodies to food antigens. Rheumatology (Oxford) 40(10):1175-9 <https://doi.org/10.1093/rheumatology/40.10.1175>

6. Sarzi-Puttini P, Comi D, Boccassini L, Muzzupappa S, Turiel M, Panni B, Salvaggio A (2000) Diet therapy for rheumatoid arthritis. A controlled double-blind study of two different dietary regimens. Scand J Rheumatol 29(5):302-7 <https://doi.org/10.1080/030097400447688>

7. Holst-Jensen SE, Pfeiffer-Jensen M, Monsrud M, Tarp U, Buus A, Hessov I, Thorling E, Stengaard-Pedersen K (1998) Treatment of rheumatoid arthritis with a peptide diet: a randomized, controlled trial. Scand J Rheumatol 27(5):329-36 <https://doi.org/10.1080/03009749850154339>

8. Nenonen MT, Helve TA, Rauma AL, Hänninen OO (1998) Uncooked, lactobacilli-rich, vegan food and rheumatoid arthritis. Br J Rheumatol 37(3):274-81 <https://doi.org/10.1093/rheumatology/37.3.274>

9. Kavanagh R, Workman E, Nash P, Smith M, Hazleman BL, Hunter JO (1995) The effects of elemental diet and subsequent food reintroduction on rheumatoid arthritis. Br J Rheumatol 34(3):270-3 <https://doi.org/10.1093/rheumatology/34.3.270>

10. Haugen MA, Kjeldsen-Kragh J, Førre O (1994) A pilot study of the effect of an elemental diet in the management of rheumatoid arthritis. Clin Exp Rheumatol 12(3):275-9

11. van de Laar MA, van der Korst JK (1992) Food intolerance in rheumatoid arthritis. I. A double blind, controlled trial of the clinical effects of elimination of milk allergens and azo dyes. Ann Rheum Dis 51(3):298-302 <https://doi.org/10.1136/ard.51.3.298>

12. Panush RS, Carter RL, Katz P, Kowsari B, Longley S, Finnie S (1983) Diet therapy for rheumatoid arthritis. Arthritis Rheum 26(4):462-71 <https://doi.org/10.1002/art.1780260403>

13. Sundqvist T, Lindström F, Magnusson KE, Sköldstam L, Stjernström I, Tagesson C (1982) Influence of fasting on intestinal permeability and disease activity in patients with rheumatoid arthritis. Scand J Rheumatol 11(1):33-8 <https://doi.org/10.3109/03009748209098111>

14. Sköldstam L, Larsson L, Lindström FD (1979) Effect of fasting and lactovegetarian diet on rheumatoid arthritis. Scand J Rheumatol 8(4):249-55 <https://doi.org/10.3109/03009747909114631>

15. Mielants H, De Vos M, Goemaere S, Schelstraete K, Cuvelier C, Goethals K, Maertens M, Ackerman C, Veys EM (1991) Intestinal mucosal permeability in inflammatory rheumatic diseases. II. Role of disease. J Rheumatol 18(3):394-400

16. Abendroth A, Michalsen A, Lüdtke R, Rüffer A, Musial F, Dobos GJ, Langhorst J (2010) Changes of Intestinal Microflora in Patients with Rheumatoid Arthritis during Fasting or a Mediterranean Diet. Forsch Komplementmed 17(6):307-13 <https://doi.org/10.1159/000322313>

17. McKellar G, Morrison E, McEntegart A, Hampson R, Tierney A, Mackle G, Scoular J, Scott JA, Capell HA (2007) A pilot study of a Mediterranean-type diet intervention in female patients with rheumatoid arthritis living in areas of social deprivation in Glasgow. Ann Rheum Dis 66(9):1239-43 <https://doi.org/10.1136/ard.2006.065151>

18. Adam O, Beringer C, Kless T, Lemmen C, Adam A, Wiseman M, Adam P, Klimmek R, Forth W (2003) Anti-inflammatory effects of a low arachidonic acid diet and fish oil in patients with rheumatoid arthritis. Rheumatol Int 23(1):27-36 <https://doi.org/10.1007/s00296-002-0234-7>

19. Fraser DA, Thoen J, Djøseland O, Førre O, Kjeldsen-Kragh J (2000) Serum levels of interleukin-6 and dehydroepiandrosterone sulphate in response to either fasting or a ketogenic diet in rheumatoid arthritis patients. Clin Exp Rheumatol 18(3):357-62

20. Denissov LN, Sharafetdinov K, Samsonov MA (1992) On the medicinal efficacy of dietetic therapy in patients with rheumatoid arthritis. Int J Clin Pharmacol Res 12(1):19-25

21. McDougall J, Bruce B, Spiller G, Westerdahl J, McDougall M (2002) Effects of a very low-fat, vegan diet in subjects with rheumatoid arthritis. J Altern Complement Med 8(1):71-5 <https://doi.org/10.1089/107555302753507195>

22. Kjeldsen-Kragh J, Haugen M, Borchgrevink CF, Førre O (1994) Vegetarian diet for patients with rheumatoid arthritis--status: two years after introduction of the diet. Clin Rheumatol 13(3):475-82 <https://doi.org/10.1007/bf02242946>

23. Kjeldsen-Kragh J, Haugen M, Borchgrevink CF, Laerum E, Eek M, Mowinkel P, Hovi K, Førre O (1991) Controlled trial of fasting and one-year vegetarian diet in rheumatoid arthritis. Lancet 338(8772):899-902 <https://doi.org/10.1016/0140-6736(91)91770-u>

24. Lanspa M, Kothe B, Pereira MR, Kesselman MM, Petrosky SN (2022) A Systematic Review of Nutritional Interventions on Key Cytokine Pathways in Rheumatoid Arthritis and Its Implications for Comorbid Depression: Is a More Comprehensive Approach Required? Cureus 14(8):e28031 <https://doi.org/10.7759/cureus.28031>

25. Philippou E, Petersson SD, Rodomar C, Nikiphorou E (2021) Rheumatoid arthritis and dietary interventions: systematic review of clinical trials. Nutr Rev 79(4):410-428 <https://doi.org/10.1093/nutrit/nuaa033>

26. Raad T, Griffin A, George ES, Larkin L, Fraser A, Kennedy N, Tierney AC (2021) Dietary Interventions with or without Omega-3 Supplementation for the Management of Rheumatoid Arthritis: A Systematic Review. Nutrients 13:3506 [https://doi.org/10.3390/nu13103506](https://www.mdpi.com/2072-6643/13/10/3506)

27. Schönenberger KA, Schüpfer AC, Gloy VL, Hasler P, Stanga Z, Kaegi-Braun N, Reber E (2021) Effect of Anti-Inflammatory Diets on Pain in Rheumatoid Arthritis: A Systematic Review and Meta-Analysis. Nutrients 13:4221 [https://doi.org/10.3390/nu13124221](https://www.mdpi.com/2072-6643/13/12/4221)

28. Wagenaar CA, van de Put M, Bisschops M, Walrabenstein W, de Jonge CS, Herrema H, van Schaardenburg D (2021) The Effect of Dietary Interventions on Chronic Inflammatory Diseases in Relation to the Microbiome: A Systematic Review. Nutrients 13:3208 [https://doi.org/10.3390/nu13093208](https://www.mdpi.com/2072-6643/13/9/3208)

29. Peltonen R, Kjeldsen-Kragh J, Haugen M, Tuominen J, Toivanen P, Førre O, Eerola E (1994) Changes of faecal flora in rheumatoid arthritis during fasting and one-year vegetarian diet. Br J Rheumatol 33(7):638-43 [https://doi.org/10.1093/rheumatology/33.7.638](https://academic.oup.com/rheumatology/article-abstract/33/7/638/1782008?redirectedFrom=fulltext&login=true)

30. Kjeldsen-Kragh J, Mellbye OJ, Haugen M, Mollnes TE, Hammer HB, Sioud M, Førre O (1995) Changes in laboratory variables in rheumatoid arthritis patients during a trial of fasting and one-year vegetarian diet. Scand J Rheumatol 24(2):85-93 <https://doi.org/10.3109/03009749509099290>

31. Peltonen R, Nenonen M, Helve T, Hänninen O, Toivanen P, Eerola E (1997) Faecal microbial flora and disease activity in rheumatoid arthritis during a vegan diet. Br J Rheumatol 36(1):64-8 <https://doi.org/10.1093/rheumatology/36.1.64>

32. Michalsen A, Riegert M, Lüdtke R, Bäcker M, Langhorst J, Schwickert M, Dobos GJ (2005) Mediterranean diet or extended fasting's influence on changing the intestinal microflora, immunoglobulin A secretion and clinical outcome in patients with rheumatoid arthritis and fibromyalgia: an observational study. BMC Complement Altern Med 5:22 <https://doi.org/10.1186/1472-6882-5-22>

33. Elma Ö, Yilmaz ST, Deliens T, Coppieters I, Clarys P, Nijs J, Malfliet A (2020) Do Nutritional Factors Interact with Chronic Musculoskeletal Pain? A Systematic Review. J Clin Med 9:702 [https://doi.org/10.3390/jcm9030702](https://www.mdpi.com/2077-0383/9/3/702)

34. Genel F, Kale M, Pavlovic N, Flood VM, Naylor JM, Adie S (2020) Health effects of a low-inflammatory diet in adults with arthritis: a systematic review and meta-analysis. J Nutr Sci 9(e37) 1-11 [https://doi.org/10.1017/jn.2020.31](https://www.cambridge.org/core/journals/journal-of-nutritional-science/article/health-effects-of-a-lowinflammatory-diet-in-adults-with-arthritis-a-systematic-review-and-metaanalysis/4A52F92292DFAC51D28965B6A090226C)

35. Schell J, Scofield RH, Barrett JR, Kurien BT, Betts N, Lyons TJ, Zhao YD, Basu A (2017) Strawberries Improve Pain and Inflammation in Obese Adults with Radiographic Evidence of Knee Osteoarthritis. Nutrients 9:949

36. Dyer J, Davison G, Marcora SM, Mauger AR (2017) Effect of a Mediterranean Type Diet on Inflammatory and Cartilage Degradation Biomarkers in Patients with Osteoarthritis. J Nutr Health Aging 21(5):562-566

37. Nelson J, Sjöblom H, Gjertsson I, Ulven SM, Lindqvist HM, Bärebring L (2020) Do Interventions with Diet or Dietary Supplements Reduce the Disease Activity Score in Rheumatoid Arthritis? A Systematic Review of Randomized Controlled Trials. Nutrients 12:2991 <https://doi.org/10.3390/nu12102991>
